# Pollinator Interactions with Yellow Starthistle (*Centaurea solstitialis*) across Urban, Agricultural, and Natural Landscapes

**DOI:** 10.1371/journal.pone.0086357

**Published:** 2014-01-17

**Authors:** Misha Leong, Claire Kremen, George K. Roderick

**Affiliations:** Department of Environmental Science Policy and Management, University of California, Berkeley, California, United States of America; University of Calgary, Canada

## Abstract

Pollinator-plant relationships are found to be particularly vulnerable to land use change. Yet despite extensive research in agricultural and natural systems, less attention has focused on these interactions in neighboring urban areas and its impact on pollination services. We investigated pollinator-plant interactions in a peri-urban landscape on the outskirts of the San Francisco Bay Area, California, where urban, agricultural, and natural land use types interface. We made standardized observations of floral visitation and measured seed set of yellow starthistle (*Centaurea solstitialis*), a common grassland invasive, to test the hypotheses that increasing urbanization decreases 1) rates of bee visitation, 2) viable seed set, and 3) the efficiency of pollination (relationship between bee visitation and seed set). We unexpectedly found that bee visitation was highest in urban and agricultural land use contexts, but in contrast, seed set rates in these human-altered landscapes were lower than in natural sites. An explanation for the discrepancy between floral visitation and seed set is that higher plant diversity in urban and agricultural areas, as a result of more introduced species, decreases pollinator efficiency. If these patterns are consistent across other plant species, the novel plant communities created in these managed landscapes and the generalist bee species that are favored by human-altered environments will reduce pollination services.

## Introduction

Human-altered landscapes are expanding globally and are often associated with declining natural habitat, non-native species, fragmentation, and transformations in structure, inputs, climate, and connectivity [1], [2], [3], [4]. These changes collectively have resulted in shifts in both spatial distributions and species diversity across many taxa including birds, mammals, reptiles, amphibians, invertebrates, and plants [5], [6]. One common driver of global change is urbanization, which in the extreme is associated with a reduction in biodiversity compared to habitats in their more natural state [7]. However, in moderately urbanized areas, the effects of urban impacts on species distribution and diversity can vary greatly and depends on region, type of change, and taxonomic group, among other factors [8], [9].

Documenting the effects of urbanization compared to natural communities has proven problematic, making predictions of community change associated with urbanization difficult. Human-altered landscapes are often associated with many non-native species which add to species diversity [6], [10], [11] but also can obscure changes in community dynamics. Thus, to assess accurately the complex impacts of land use change on ecological communities, one must look beyond species richness to investigate ecological processes themselves. Ecological processes are the links between organisms in a functioning ecosystem, and are critical in understanding how altered biodiversity can lead to changes in ecosystem functioning [12].

Global environmental change has been found to have a wide variety of impacts on ecological processes in different systems [13]. Pollinator-plant relationships in particular are found to be particularly vulnerable to land use change, resulting in decreases in interaction strength and frequency [14]. Pollination services are crucial ecosystem processes in natural systems, but also in agricultural and urban areas [15]. Bees provide the majority of animal-mediated pollination services on which it is estimated 87.5% of flowering plants depend [16]. The value of pollination in agriculture is estimated at $200 billion worldwide [17], largely due to many foods that are essential for food security and a healthy human diet, including numerous fruits, vegetables, and nuts that require bee pollination. As urban areas expand, there has been increasing interest in urban agriculture to ensure food security and access to healthy foods for growing populations, and these systems also depend on pollination. For example, Kollin [18] estimated that the economic value of urban fruit trees (many of which require pollination) to be worth $10 million annually in San Jose, California.

Despite the important role of pollinators and concerns about bee declines [19], [20], there remain many uncertainties regarding the impact of land use change on pollinators [21]. Urbanization has resulted in more interfaces with both natural and agricultural landscapes, creating new transitional zones of peri-urbanization [22]. While there has been extensive pollinator research in agricultural and natural systems [23], [24], [25], [26], [27], less attention has focused on pollination in neighboring urban areas and how the changing landscape has impacted pollination [9], [28]. In addition, very few studies of urban areas have looked beyond changes in bee diversity to understand explicitly the effect of urbanization on pollinator-plant interactions [10], [29], [30].

Here, we investigate the effect of land use change on pollinator-plant ecosystem processes. We make use of a “natural experimental design” in which urban, agricultural, and natural areas intersect. Bees visit flowers for both pollen and nectar resources, and floral visitation is a commonly used as an index of pollination services. However, depending on the flower, certain bee groups are much more effective pollinators than others [9], [21], [31]. Thus, while visitation is important, it alone does not definitively indicate whether pollination services were received by the plant [32]. When pollen is limited by other factors, consequences for plant fitness can include failure to set seed, production of smaller fruits, and even complete lack of reproduction [33], [34]. By looking at rates of bee visitation and comparing this with other measures of plant fitness, such as seed set, we can develop a more complete understanding of how shifts in bee distributions between areas that differ in land use are impacting pollination services.

To study the impact of changing land use on pollinator-plant interactions, we focus on bee pollination of a widespread plant, yellow starthistle (*Centaurea solstitialis*), a common weed found in natural, agricultural, and urban habitats. Using standardized observations of floral visitation and seed set measurements of yellow starthistle, we test the hypotheses that increasing urbanization decreases 1) rates of bee visitation, 2) viable seed set, and 3) the efficiency of pollination (relationship between bee visitation and seed set). In addition to contributing to a better understanding of how change in landscape use, particularly urbanization, affects pollination-plant interactions, the study illustrates the importance of use of neighboring lands for pollination services.

## Methods

### Ethics Statement

No protected species were sampled in this field study. Permits and approval were obtained for field observations on public land from the East Bay Regional Park District, Contra Costa County Flood Control and Water Conservation District, and the Los Vaqueros Reservoir.

### Study System

**Figure 1:**
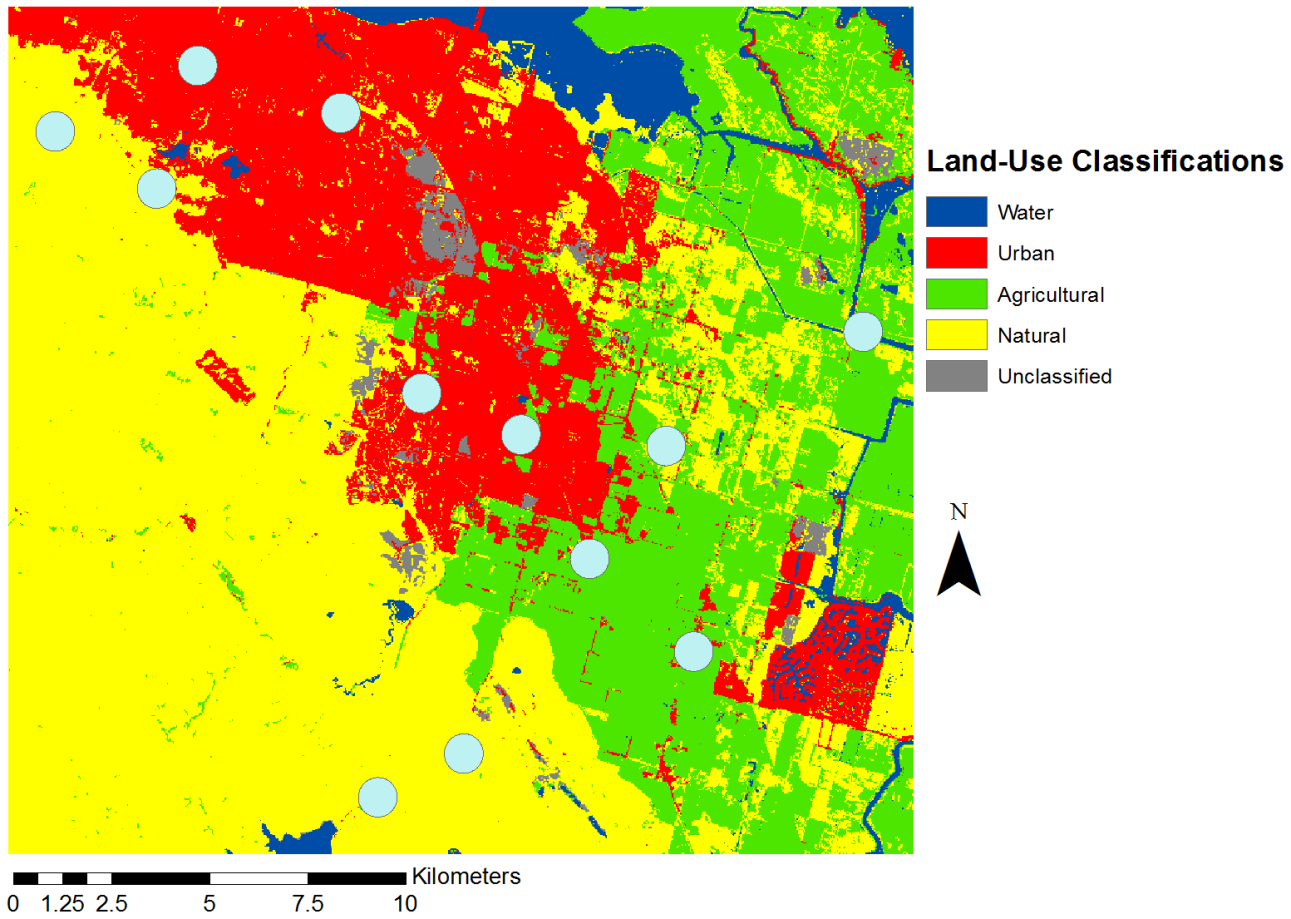
Map of study area and locations of plots in East Contra Costa County, California. Light blue dots represent a 500(green), urban (red), and natural (yellow) land use types.

Our study system was located around Brentwood, in east Contra Costa County, California, where natural, agricultural, and urban areas intersect with each other within a 20×20 km region (Figure 1). A county water district (Los Vaqueros Watershed), regional park district (East Bay Regional Parks: Black Diamond Mines, Round Valley, and Contra Loma), and California state park (Mount Diablo) all fall within the region, leaving large areas of land protected from development. This protected (hereafter referred to as “natural”) land consists mainly of grasslands and oak woodlands, some portions of which are managed for grazing. East Contra Costa County has had a farming community presence since the late 19^th^ century. The agricultural areas of Brentwood, Knightsen, and Byron mostly consist of orchards (cherries, stone fruit, grapes and walnuts), corn, alfalfa, and tomatoes [35]. A housing boom in the 1990s led to massive residential growth in the area. The city of Brentwood has grown from less than 2500 people in the 1970s to over 50,000 today (2010 U.S. Census), and nearby Antioch has now over 100,000 residents (2010 U.S. Census).

We selected 12 sites dominated by yellow star thistle in a stratified design to span the different land use types (Figure 1). Yellow starthistle (*Centaurea solstitialis*) is a common weedy plant that forms homogenous flowering patches in grassy areas throughout this region. Many different bee taxa in a range of functional groups and size classes have been observed to visit yellow starthistle [36], in part because it flowers late in the season relative to other floral resources [37]. Despite being considered a serious introduced weed, yellow starthistle is unusual as an invasive species in that it depends on animal pollinator visits in order to set seed [38].

Within each site we selected a 50 m×50 m plot such that each plot was at least 2 km away from all others, a distance larger than the maximum assumed typical bee foraging ranges [39]. Although certain bee species have been recorded foraging as far as 1400 m [40], most bees in this type of habitat have nesting and foraging habitat within a few hundred meters of each other [39], [40], [41]. Within each plot we estimated number of flowering yellow starthistle blooms by randomly placing 10, 1 m×1 m quadrats and counting the number of flowering blooms in each. We also measured the spatial area of yellow starthistle patches within each 50 m×50 m plot to obtain an estimate of total flowering blooms within each plot. We categorized total blooms/plot on a log scale: <10^3^ (Category 1), 10^3^–10^4^ (Category 2), and >10^4^ (Category 3).

Using NOAA’s 2006 Pacific Coast Land Cover dataset (developed using 30 meter resolution Landsat Thematic Mapper and Landsat Enhanced thematic Mapper satellite imagery, USGS Products), a 500 m buffer (representing estimated bee foraging ranges [39], [40], [41]) was created around each plot, and the number of pixels classified as agricultural, urban, natural, water, or bare land was extracted. These categories were obtained by lumping finer categories in NOAA’s classification scheme using the following definitions: Urban–“High Intensity Developed”, “Medium Intensity Developed”, “Low Intensity Developed”, and “Developed Open Space”; Agricultural–“Cultivated”, “Pasture/Hay”; Natural– “Grassland”, Deciduous Forest”, “Mixed Forest”, “Scrub/Shrub”. Each plot was classified as a proportion of each of the 3 different land use categories, as well as for the category that was dominant. By this latter measure, of our 12 sites, 4 of each were classified as “urban”, “agricultural”, and “natural”.

### Bee Visitation

We observed visits by all bee species to yellow starthistle at all sites 3 times (AM, mid-day, and PM) for a 30 min period for a total of 90 min of total observation time per site within the same 2 wk period in August 2011. AM was defined as being between the hours of 9∶30–11∶30, Mid-Day as between 11∶30–13∶30, and PM as between 13∶30–15∶30. All observations were conducted by the same individual (ML) to avoid sampling biases. Also recorded at each observation period were approximate number of blooms, and wind and temperature simultaneously (using a Kestrel 3000 Pocket Weather Meter). Bees were not netted for later identification as we did not want to interfere with visitation to starthistle during this study. Instead, we used a modified protocol of citizen scientist observation surveys [42] with 15 expected bee morphotypes (Table 1) that correspond to 30 possible genera known to occur in the region (Leong, unpublished data). The observer slowly walked through the yellow starthistle patch, and upon reaching patch edge, returned on a path at least 3 m away from the previous, and recorded the morphotype classification of all bee visitors within 1.5 m on either side of the transect.

**Table 1:** The 15 bee morphotypes observed and their associated genera and species in East Contra Costa County, California.

Morphotype	Possible Species
Honey bee	*Apis mellifera*
Bumblebee	*Bombus spp.*
Carpenter bee	*Xylocopa spp.*
Hairy leg bee, medium	*Melissodes spp., Anthophora spp., Eucera spp., Peponapis spp., Exomalopsis spp., Diadasia spp.*
Hairy leg bee, large	*Svastra spp.*
Green sweat bee	*Agapostemon texanus*
Striped sweat bee, medium	*Halictus ligatus, Halictus spp.*, >0.5 cm
Striped sweat bee, small	*Halictus tripartitus, Halictus spp.*, <0.5 cm
Small dark bee, rounded tip	*Lasioglossum spp.*
Small dark bee, shield tip	*Ceratina spp.*
Striped hairy belly bee, small	*Ashmeadiella spp., Megachile spp.*, <0.5 cm
Striped hairy belly bee, medium	*Megachile*, >0.5 cm, <1.5 cm
Striped hairy belly bee, large	*Megachile*, >1.5 cm)
Wasp-like hairy belly bee	*Dianthidium app., Anthidium spp.*
Cuckoo bee	*Sphecodes spp., Nomada spp., Nomia spp., Calliopsis spp.*

### Seed Set

Yellow starthistle (Asteraceae) has composite flowers, which are aggregations of anywhere from 20–80 florets [43]. At each site, 12 yellow star thistle buds were randomly selected from different plants and covered with a mesh bag. Yellow starthistle blooming cycles have been described in detail in other publications [43]. We selected buds at stage BU-4 [43], when buds had no yellow petals exposed, but had well-developed straw-colored spines. When in full flowering, 10 bags were opened for a 4 hour period from 10 am to 2 pm, while 2 were kept closed as controls to verify that self-pollination was not occurring. At the opening and re-closing of the bags, the number of florets that had their stigmas extended (and thus, available for pollination) were counted. Later, when flowers were fully mature (dry and straw-colored), seed heads were collected, and later dissected in the lab. Viable and non-viable seeds in yellow starthistle seed heads are easily distinguishable based on color and shape [38]. Because yellow starthistle requires pollination to produce viable seeds (also confirmed by our controls), non-viable seeds represent pollen limitation occurring during the 4-hour period that the flowers were exposed to pollinators. All seeds were counted to compare ratios of viable to non-viable seeds. Any seed predation was noted, and when possible, the seed predator was identified.

### Analyses

All analyses were done in R 2.15.1 (R Development Core Team, 2011). Because each site had an AM, Mid-Day, and PM observation event, there were a total of 36 observation events, each with unique wind and temperature recordings, and visit observations of the 15 bee morphotypes. From these, we calculated the total number of bee visitors, total number of bee morphotypes, Shannon diversity of morphotypes, and morphotype evenness. Shannon diversity and evenness were calculated using the R package *vegan* [44]. The spatial autocorrelation of all bee visitor response variables (each morphotype abundance, total abundance, morphotype richness, diversity, and evenness) was assessed by Mantel tests in R package *ade4* [45], using the average values for each time of day at each site. Spatial autocorrelation was not detected (*p≥*0.14).

To test for the effect of land use type on each of the response variables we used a generalized linear mixed model using the R package *lme4* [46]. We designated land use type, bloom category of flowering patch, observation time period, wind, and temperature as fixed effects and site as a random effect. Natural land use and AM observation time period were the model baselines for the categorical variables of land use type and observation time. Shannon diversity and evenness were fit with Gaussian distributions while all other variables were fit with Poisson distributions.

In comparing the ratios of viable seeds to total seeds vs. the ratio of viable seeds to counted stigmas, we found that there was a strong correlation between these metrics. To look at the effect of land use type on seed-set, we therefore decided to utilize the ratio of viable seeds to total seeds in each seed head that did not experience seed predation, because of error in counting the number of stigmas (in some cases, we had slightly more viable seeds than counted number of stigmas, suggesting errors in this measurement). We then used a generalized linear mixed model fit with a Binomial distribution, with land use type as a fixed effect and site as a random effect.

Finally, we tested for an effect of floral visitor observations on yellow starthistle seed set at each site. We averaged the number of visits from each morphotype across temporal observation events at the same site. Morphotypes that averaged at least one visit per 30 minute observation window were included as fixed effects in a linear mixed model fit with a binomial distribution, with site as a random effect and the ratio of viable to total seeds as the response variable. We also modeled the effects of total bee visitation, morphotype richness, and morphotype diversity on seed set ratios.

Data on bee visitation rates for all observation events and seed set ratios for each plant are available from the Dryad Digital Repository: http://dx.doi.org/10.5061/dryad.b5np1 [47].

## Results

### Bee Visitation and Land Use

**Figure 2:**
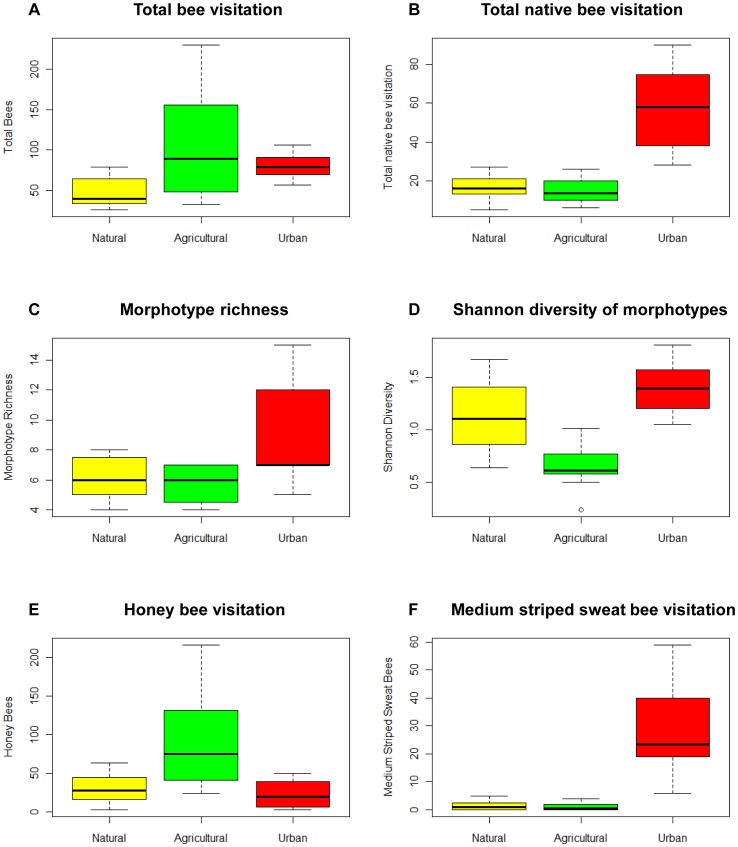
Box plots of bee visitation response variables in natural, agricultural, and urban sites. Bee morphotype visitation data and calculated community metrics were collected in East Contra Costa County, California.

A total of 2816 total bee visits were recorded, representing 15 bee morphotypes. Total bee visitation was significantly higher in urban and agricultural areas with respective effect sizes (± standard errors) of 0.885±0.26 (p = 0.0007) and 0.813±0.22 (p = 0.0002) (Figure 2, Tables 2 & 3). The effect of land use type on visitation rates when analyzed separately for each bee morphotype, was a significant variable for 6 of 15 morphotypes. Bloom category, time of observation, wind, and temperature were only occasionally significant in some of the models.

**Table 2:** Statistical output table for response variables having significant relationships with the agricultural land use type.

Response Variable	Effect size	Standard error	p- value
Total bee visitation	0.813	0.22	0.0002
Honey bees	1.26	0.33	0.0002
Shield-tipped small dark bees	1.83	0.78	0.02
Medium striped hairy belly bees	1.53	0.62	0.01
Morphotype Shannon diversity	−0.488	0.193	0.009
Morphotype evenness	−0.264	0.086	0.002

Bee morphotype visitation data and calculated community metrics were collected in East Contra Costa County, California. Significant relationships with the agricultural land use type were calculated based on generalized linear mixed models with land use type, bloom category of flowering patch, observation time period, wind, and temperature as fixed effects and site as a random effect. The natural land use type and morning (AM) observation time period were the model baselines for the categorical variables of land use type and observation time. Shannon diversity and evenness were fit with Gaussian distributions while all other variables were fit with Poisson distributions.

**Table 3:** Statistical output table for response variables having significant relationships with the urban land use type.

Response Variable	Effect size	Standard error	p- value
Total bee visitation	0.885	0.26	0.0007
Native bee visitation	1.389	0.273	<0.0001
Medium striped sweat bees	3.213	0.268	<0.0001
Small striped sweat bees	1.74	0.53	0.001
Small striped hairy belly bees	1.055	0.536	0.04
Morphotype richness	0.369	0.100	0.06

Bee morphotype visitation data and calculated community metrics were collected in East Contra Costa County, California. Significant relationships with the urban land use type were calculated based on generalized linear mixed models fit with Poisson distributions with land use type, bloom category of flowering patch, observation time period, wind, and temperature as fixed effects and site as a random effect. The natural land use type and morning (AM) observation time period were the model baselines for the categorical variables of land use type and observation time.

Agricultural sites (Table 2) had the highest total bee visitation; 62% of total bee observations were honey bees (*Apis mellifera*), which were observed significantly more often in agricultural, than managed or urban sites (effect size±SE = 1.26±0.33, p = 0.0002). Agricultural sites also had significantly higher visitation rates from shield-tipped small dark bees (effect size±SE = 1.83±0.78, p = 0.02) and medium striped hairy belly bees (effect size±SE = 1.53±0.62, p = 0.01). However, agricultural sites, compared to natural and urban sites, had significantly lower morphotype Shannon diversity (effect size±SE = −0.488±0.193, p = 0.009) and morphotype evenness (effect size±SE = −0.264±0.086, p = 0.002).

Visitation by native bees (here measured as visitation by non-honey bees, although there are a few other non-native species that may be included within the other morphotypes) was highest in urban sites (Table 3) compared to those in the other land use types (effect size±SE = 1.389±0.273, p<0.0001). Medium and small striped sweat bees were the most abundant groups after honey bees, which made up 12% and 7% of total bee observations respectively. When analyzed by morphotype, urban sites had the highest visitation levels from medium striped sweat bees (effect size±SE = 3.213±0.268, p<0.0001), small striped sweat bees (effect size±SE = 1.74±0.53, p = 0.001), and small striped hairy belly bees (effect size±SE = 1.055±0.536, p = 0.04). Urban areas had higher morphotype richness (effect size±SE = 0.369±0.199, p = 0.06), but this effect was not significant.

None of the morphotypes were observed significantly most often in natural sites, although 2 of 3 sites where bumblebees were observed were natural sites.

**Figure 3:**
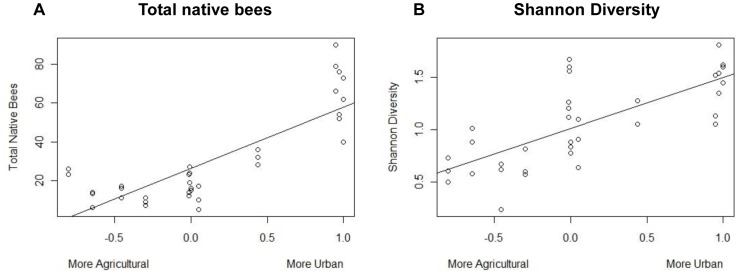
Bee visitation response variables as a function of surrounding anthropogenic land use. Bee morphotype visitation data and calculated community metrics were collected in East Contra Costa County, California. To examine in more detail the effect of anthropogenic land use on bee visitation, we created a continuous variable for land use with an index ranging from agricultural to urban land use based on proportional area of each type within a 500-axis moves from left to right, sites go from being more agricultural to more urban.

To examine in more detail the effect of land use on bee visitation, we created a continuous variable for land use with an index ranging from agriculture to urban use based on proportional area of each type within a 500 m radius. We then used this measure of land use to assess the response of total bee visitation, native bee visitation, morphotype richness, evenness, and Shannon diversity using previously described mixed model techniques. We found that while there was no significant effect of land use on total bee visitation, native bee visitation observations increased with more surrounding urban area (effect size±SE = 0.963±0.22, p<0.001). We also saw the same effect of increasing surrounding urban area on morphotype richness (effect size±SE = 0.27±0.12, p = 0.02), Shannon diversity (effect size±SE = 0.55±0.144, p<0.01) and evenness (effect size±SE = 0.237±0.069, p<0.01) (Figure 3, Table 4).

**Table 4:** Statistical output for response variables having significant relationships with the gradient of agricultural to urban land use.

Response Variable	Effect size	Standard error	p- value
Native bee visitation	0.963	0.22	<0.01
Morphotype richness	0.27	0.12	0.02
Morphotype Shannondiversity	0.55	0.144	<0.01
Morphotype evenness	0.237	0.069	<0.01

To examine in more detail the effect of anthropogenic land use on bee visitation, we created a continuous variable for land use with an index ranging from agriculture to urban land use based on proportional area of each type within a 500 m radius. Generalized linear mixed models were created with this calculated anthropogenic land use metric, bloom category of flowering patch, observation time period, wind, and temperature as fixed effects and site as a random effect. The morning (AM) observation time period was the model baseline for the categorical variable of observation time. Shannon diversity and evenness were fit with Gaussian distributions while all other variables were fit with Poisson distributions.

### Seed Set

**Figure 4:**
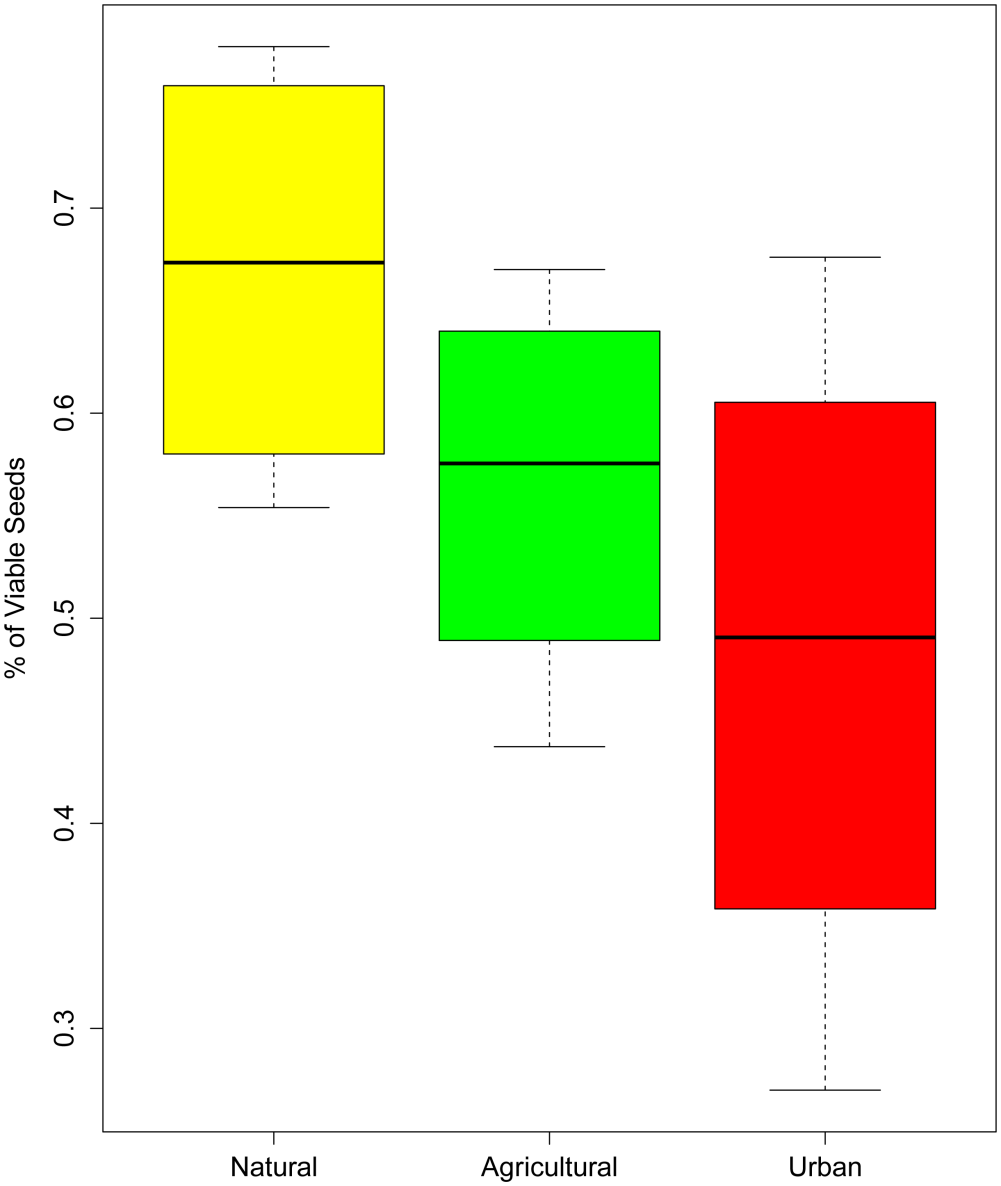
Box plot demonstrating the effect of land use type on percentage of viable seeds. Yellow starthistle seed heads were collected in East Contra Costa County, California and dissected in the lab after maturity. We calculated significance using a generalized linear mixed model fit with a binomial distribution, with land use type as a fixed effect and site as a random effect. With natural sites as the baseline, urban areas had significantly lower rates of seed set (effect size±SE = −0.756±0.371, p = 0.042).

Natural sites had the highest average rates of seed set, and urban areas had the lowest (effect size±SE = −0.756±0.371, p = 0.042, Figure 4), in direct contrast to the pattern found with floral visitation where urban sites had the highest rates of native bee visitation and natural sites had the lowest. In total 140 yellow starthistle seed heads were collected and dissected; 4 lost mesh bags in the field and were eliminated from the study. Of these, 43% of the collected seed heads experienced some type of seed damage, largely due to biological control efforts in the area involving tephritid flies and weevils. Seed predation decreased with amount of surrounding agricultural area (simple linear regression, p<0.01). Of the 79 seed heads that were intact, 73 had received the 4-hour treatment of being exposed to pollination. Only 6 flowers in the control group which were never exposed to pollinators experienced no predation. Of those, 3 had no viable seeds, and 3 had 4.7%, 8.3%, and 20% viable seeds respectively.

### Bee Visitation and Seed Set

**Figure 5:**
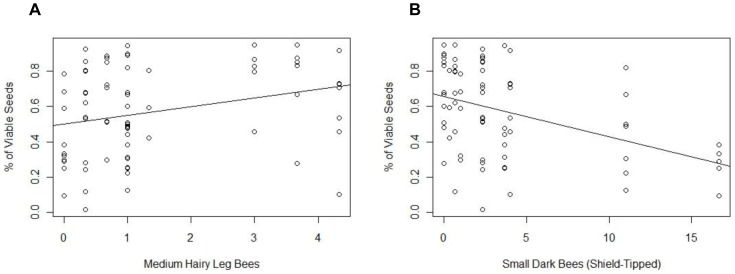
Correlation between the percentage of viable seeds in each yellow starthistle seed head and the average number of site visits by morphotype. Bee morphotype visitation data, calculated community metrics, and yellow starthistle seed heads were collected in East Contra Costa County, California. Bee morphotypes that averaged at least one visit per 30 minute observation window were included as fixed effects in a linear mixed model fit with a binomial distribution, with site as a random effect and the ratio of viable to total seeds as the response variable. Medium hairy leg bees (effect size±SE = 0.284±0.069, p<0.001) and shield-tipped small dark bees (effect size±SE = −0.155±0.051, p = 0.002) had significant effect sizes in the model. Regression lines were added to illustrate relationships.

Of the 8 morphotypes that averaged at least one visit per 30 minute observation period, 3 exhibited significant relationships between visitation abundance at a site and seed set, although there was no significant relationship between site seed set and total bee visitation, morphotype richness, or morphotype diversity. Increased seed set ratios correlated with sites that had more visitation from medium hairy leg bees (effect size±SE = 0.284±0.069, p<0.001) and to a smaller extent, round-tipped small dark bees (effect size±SE = 0.127±0.074, p = 0.04), despite there not being significant relationships between land use type and either of these bee groups. However, visitation by shield-tipped small dark bees (effect size±SE = −0.155±0.051, p = 0.002) had a significant negative effect on proportion of viable seeds (Figure 5).

## Discussion

Our results show that rates of bee visitation and seed set vary among urban, agricultural, and natural landscapes, demonstrating the importance of land use in the dynamics of plant-pollinator interactions. We suggest that these effects are at least in part explained by floral availability, a vital bee resource, which can be highly variable among different land use types. For example, in August there are few plants in flower besides yellow starthistle in the natural areas of Contra Costa County, California, whereas in urban and agricultural areas there are many exotic plants and supplementary inputs available (personal observation). From pan-trapping of bee specimens in the region (Leong, unpublished data), we know that total bee abundance is highest in the spring in natural areas. However, towards the end of the summer when yellow starthistle is in flower, there is little difference in collected bee abundance between human-altered landscapes and natural areas, and human-altered areas may even exhibit overall higher bee abundance.

Our results of bee visitation to yellow starthistle support this pattern. Agricultural areas have large populations of managed honey bee colonies, so one would predict visitation to yellow starthistle by honey bees to be positively associated with surrounding agricultural land use. By contrast for native bees (total bee visitation excluding honey bees), the highest rates of visitation to yellow starthistle were in sites with more surrounding urban land use. Urban gardens have many exotic plants, often selected for aesthetic purposes, many of which are in flower later in the season than most California native plants. In addition, many of the plants in urban areas both directly and indirectly receive supplementary resources, particularly water, that further extend their flowering time. Even though agricultural areas also have supplementary resources, the main crop in flower in East Contra Costa County later in the season is maize, which is wind-pollinated. There may be multiple impacts of exotic plants in urban areas. By filling the phenological flowering gap [48] noted above, they may help attract even larger populations of bees into the urban landscape. In addition, bees in urban sites may be behaviorally more likely to visit non-native plants due to the increased encounters they have with novel plants [49].

In agricultural and natural landscapes, a positive correlation between pollinator visitation and seed set is typical [50]. Surprisingly in our system, in human-altered landscapes, higher total observed bee visitation did not result in higher proportions of seed set, as would be expected. In fact, urban areas, despite receiving the highest rates of native bee visitation, exhibited the lowest rates of seed set. Conversely, natural areas, which received the lowest amount of total bee visitation, had the highest rates of seed set.

We suggest 2 possible explanations for this discrepancy between pollinator visitation and rates of seed set: 1) pollinator efficiency; and/or 2) the composition of the local flowering community. Depending on the plant, certain pollinator species are much more effective than others [51]. For example, *Osmia, Habropoda,* and *Apis*, have been found to produce varying amounts of seed set as a result of a single visit to blueberry, but these results vary slightly depending on the blueberry variety [52]. In the case of yellow starthistle, it is likely that the most frequent visitors are perhaps not the most efficient. When we directly compared average seed set at each site against visitation rates, we found a significant positive association with the medium hairy leg bees. The medium hairy leg bee morphotype includes those species which fall in both the Tribes Emphorini and Eucerini. Emphorini are known to largely be oligolectic (Michener 1999), meaning they specialize on certain plant groups, which theory suggests would make them more efficient pollinators than generalists [51].

The medium hairy leg bee morphotype was not significantly associated with any of the land use typesIt was also the only group that was observed most frequently during morning (AM) sampling, perhaps reflecting a difference in when yellow starthistle is most receptive to pollination. Despite the overwhelming abundance of honey bees in agriculture areas, we did not observe higher seed set in those regions, consistent with the observation that honey bees can be poorer pollinators than other species [53], [54].

It is also important to note that this study used a morphotype classification, and there may be multiple species that fit within the same morphotype that provide varying degrees of pollination services [55]. It is possible there are rare, but highly efficient, pollinators that were rarely observed during the sampling period, or were lumped together with a more frequently observed morphotype.

An alternative explanation for the lack of an association between floral visitation and seed set is that higher plant diversity in urban and agricultural areas may decrease pollinator efficiency. Previous research has shown that invasive alien plants can have a negative effect on native plant communities by acting as attractors for pollinators, or decreasing pollinator efficiency by providing a wider range of resources for pollinators to visit, with the consequence that visitors transfer pollen from non con-specifics, potentially clogging stigmas and reducing pollination success [56], [57], [58]. In this case, our target plant, yellow starthistle is indeed considered an invasive alien plant, but the hypothesis of it being in a novel diverse community could lead to a similar effect on the frequency and quality of pollination services that it receives. In sites where there are many other potential plants to visit and accompanying decreased floral fidelity leading to diverse pollen loads, one predicts decreased pollinator efficiency. Abundant sources of exotic plant pollen could occur in areas where there is a greater diversity of nearby plants for pollinators to visit. This explanation might account for the observation that shield-tipped small dark bees were negatively correlated with seed set.

We selected yellow starthistle as the target plant for this study because of its ubiquitous distribution, reliance on pollination, and its attraction for a wide set of visitors; it is also a highly invasive and undesirable plant [59]. Previous research on yellow starthistle has found that its invasion can be facilitated other non-native pollinator species such as the honey bee, *Apis mellifera*, and the starthistle bee, *Megachile apicalis* [36], [38], which is included in the medium striped hairy belly bee morphotype. However, the abundance of bees in both of these 2 morphotypes were most closely associated with agricultural areas, which did not have the highest rates of seed set as would be predicted by visitation alone.

Our results indicate clearly that bee visitation in human-altered landscapes can be higher than that in comparable natural areas, especially towards the end of the flowering season when there are few resources available in natural landscapes. Because the response of bee visitors to land use change depends on species-specific requirements and these pollinators also have variable effects on plants, understanding the effect of land use change on pollination services requires knowledge not only of which pollinator groups shift to the human-altered landscapes, but also the rate of pollination that those groups have on the plant species in those landscapes. Future research will benefit from looking at a wider range of plants with a different range of target pollinators and that flower earlier in the year to better tease out these hypotheses. If the patterns of bee visitation and seed set that we observed are indeed consistent across other plant species, the novel plant communities created in these human-altered landscapes and the generalist bee species that are favored in such landscapes will lead to a reduction in overall pollination services.
